# LncRNA NCAL1 potentiates natural killer cell cytotoxicity through the Gab2-PI3K-AKT pathway

**DOI:** 10.3389/fimmu.2022.970195

**Published:** 2022-09-28

**Authors:** Chao Niu, Min Li, Yongchong Chen, Xiaoying Zhang, Shan Zhu, Xin Zhou, Lei Zhou, Zhaozhi Li, Jianting Xu, Ji-fan Hu, Yufeng Wang, Jiuwei Cui

**Affiliations:** ^1^ Department of Cancer Center, First Hospital, Jilin University, Changchun, China; ^2^ Department of Translational Medicine, First Hospital, Jilin University, Changchun, China; ^3^ Cancer Institute, First Hospital, Jilin University, Changchun, China; ^4^ Stanford University Medical School, Veterans Affairs (VA) Palo Alto Health Care System, Palo Alto, CA, United States

**Keywords:** noncoding RNA, natural killer cell, epigenetics, cytotoxicity, immune therapy

## Abstract

Natural killer (NK) cells perform immune surveillance functions in tumors. The antitumor effects of NK cells are closely related to tumor occurrence and development. However, the molecular factors that determine NK cell antitumor activity remain to be characterized. In the present study, we identified a novel long noncoding RNA (lncRNA), NK cell activity-associated lncRNA 1 (NCAL1), and investigated its function in NK cells. NCAL1 was primarily located in NK cell nuclei, where it functioned by activating Gab2, a scaffold protein with an essential role in immune cells. Gab2 positively regulated the killing activity of NK cells. Mechanistically, NCAL1 upregulated Gab2 epigenetically by binding to the *Gab2* promoter, which decreased methylation, recruited the transcription factor Sp1, and increased H3K4me3 and H3K27ac levels in the Gab2 promoter. Furthermore, NCAL1 enhanced the cytotoxicity of NK cells toward tumor cells through the Gab2-PI3K-AKT pathway. Thus, NCAL1 potentiates NK cell cytotoxicity and is a promising therapeutic target to improve NK cell therapy.

## Introduction

Natural killer (NK) cells are important immune cells involved in innate immunity. These cells act as the first line of defense against cancer cells and tumor virus infection (1, 2). Further, NK cells can directly kill tumor cells. Because of nonspecific effects, the natural killing activity of NK cells does not require antigen participation and has no major histocompatibility complex restrictions (3, 4). NK cells also play potent immunomodulatory roles by interacting with other immune cells to regulate the immune state and function in the body (1, 5). Clinical studies demonstrated that adoptive immunotherapy using NK cells has potential applications for malignant tumors and exerts effects on various solid tumors and hematological malignancies (6). Therefore, NK cell therapy is a promising immunotherapy strategy for treating various cancers (7, 8). Although NK cell activity controls tumor growth, NK cells are susceptible to many immunosuppressive mechanisms in the tumor microenvironment (9, 10). Ways to improve the antitumor effect of NK cells should be a focus of research on NK cell therapy in the future (8).

Long noncoding RNAs (lncRNAs) are noncoding RNAs that are more than 200 nucleotides long (11). Most transcripts produced by mammalian genome sequences are lncRNAs (12). Previous studies showed that lncRNAs play important roles in epigenetic regulation, the cell cycle, cell differentiation, and many other activities. Thus, lncRNAs have recently gained attention in genetic research (13–15). Although research on lncRNAs has made rapid progress, the functions of most lncRNAs remain unclear (16), particularly in NK cells, in which only two lncRNAs have been reported (17, 18). lnc-CD56 positively correlates with CD56 expression in primary human NK cells and differentiates NK cells from human hematopoietic progenitor cells (17). lncRNA-GAS5 promotes NK cell activity against gastric cancer by reducing miR-18a (18). Determining lncRNA functions in NK cells would improve our understanding of NK cell-mediated antitumor mechanisms.

We previously found that the histone deacetylase inhibitor, valproic acid, can reduce NK cell cytotoxicity (19). In this study, we performed next-generation sequencing (NGS) on activated human NK cells treated with valproic acid to identify lncRNAs that regulate NK cell activity. We identified a previously uncharacterized lncRNA, NK cell activity-associated lncRNA 1 (NCAL1), as a potential factor that regulates NK cell-mediated cytotoxicity. We aimed to investigate the mechanism underlying the role of NCAL1 in NK cells.

## Materials and methods

### Cell culture

Peripheral blood mononuclear cells (PBMCs) were isolated from the blood of six healthy donors using Ficoll (cat: 1114547; Axis-Shield PoC AS, Oslo, Norway) gradient density centrifugation. This procedure was approved by the Ethics Committee of the First Hospital of Jilin University (2017–022). All participants provided informed consent to participate in this study.

Primary NK cells were isolated from human PBMCs using a MACSxpress NK Cell Isolation Kit (cat: 130-098-185; Miltenyi Biotec, Bergisch Gladbach, Germany) and were cultured in Aly505 medium (cat: 01400P10; Cell Science & Technology Institute, Inc., Yamagata, Japan) containing 10% autologous serum and 600 IU/mL interleukin-2 (cat: 130-097-743; Miltenyi Biotec). B cells, T cells, and γδ T cells were sorted with FACS Aria II (BD Biosciences, San Jose, CA, USA).

NK92MI cells (cat: CL-0533; Procell Life Science & Technology Co., Ltd., Wuhan, China) were cultured in α-minimum essential medium (cat: 1749161; Gibco, Grand Island, NY, USA) containing 2 mM L-glutamine (cat: 25030081; Gibco), 1.5 g/L sodium bicarbonate (cat: S5761; Sigma-Aldrich, St. Louis, MO, USA), 0.2 mM inositol, 0.1 mM β-mercaptoethanol (cat: M6250, Sigma-Aldrich), 0.02 mM folic acid (cat: F8758, Sigma-Aldrich), 12.5% horse serum (cat: 16050122; Gibco), and 12.5% fetal bovine serum (cat: SFBE; Natocor, Córdoba, Argentina). Gab2-overexpressing NK92MI cells were cultured with 5 μM of the PI3K inhibitor pictilisib (cat: S1065; Selleckchem, Houston, TX, USA) for 24 h.

### Rapid amplification of cDNA ends (RACE) of NCAL1

Full-length NCAL1 was obtained using 3′ and 5′ RACE, which was performed using a Marathon cDNA Amplification Kit (cat: 634913; TaKaRa, Shiga, Japan). The primers used for RACE are listed in **Table 1**.

**Table 1:** Primers used in the study.

Experiments	Target		Sequence (5′-3′ direction)
qPCR	NCAL1	sense	GCAAGAAACCTCAGGACTTGAG
		antisense	CAACACTCTGGTGGTAGTCTGA
	*Gab2*	sense	TCAGCAGAGACCGCCAATCAGT
		antisense	GGTACTCGTAGGTCTCACAGGA
ChIP qPCR for H3K27ac and H3K4me3	I	sense	CTTTCAGCCGGAACCTGACC
		antisense	GTCGACGGGGAAGGGATACTA
	II	sense	CGAGAAGAAGTTGAGGCGCT
		antisense	TTTAGTTCACGAGCAGACCG
	III	sense	GCATGTGCAGCCCTTGTATT
		antisense	CCTGTGAGAGACGAACGGAA
ChIP qPCR for Sp1	I	sense antisense	CTGTGTTTTCCCAGAGGCAGT
			CCGGAGCCTTTGCTTTTGTAG
	II	sense antisense	CGTAGTAGGTCCGTCTCCTC
			ATCGATTTTTCACCGAGCGT
	III	sense antisense	TGACCCGCAGAATGTATCGG
			AGGATACAGAAAGCCACCGC
RAT qPCR	I	sense	CCCCCAGTCCAATCACTGAC
		antisense	GTGCACTGTGGCTTTGAGTG
	II	sense	TAGATCCAGGGGACTGACCG
		antisense	ATTCAATGGGGAACCGTGCT
	III	sense	GAGCTCGGAATAGACCCTGC
		antisense	CAGAGGCCAGAAGTCCCTTG
	IV	sense	ACATGACTGGAAGCCTCTGC
		antisense	GCAGCAGCTACTTTGGGGTA
	V	sense	ACTTGGTGGTGGTTTATGCAGT
		antisense	AGTAGGTTTCCACTGGAGCCG
BSP	CpG island of Gab2	sense	GTTTTAGGAGAAAGCGAGATTTC
		antisense	AAACAACTACAACAAAAAAACCGAC
RAT	I	antisense	GACTTGTCCAGGTGCTACTC
	II	antisense	TGCTCTTCCCATCAGAGATGC
	III	antisense	TCAGACTACCACCAGAGTGTTG
siRNA	si-NC	sense	UUCUCCGAACGUGUCACGUTT
		antisense	ACGUGACACGUUCGGAGAATT
	si-NCAL1-1	sense	GCAAGAAACCUCAGGACUUTT
		antisense	AAGUCCUGAGGUUUCUUGCTT
	si-NCAL1-2	sense	CCAGAGUGUUGAGUCUCAUTT
		antisense	AUGAGACUCAACACUCUGGTT
	si-*Gab2*-1	sense	ACCUCAAACCUGAUCGGAATT
		antisense	UUCCGAUCAGGUUUGAGGUTT
	si-*Gab2*-2	sense	CACUUGACCUGAGGAACAATT
		antisense	UUGUUCCUCAGGUCAAGUGTT
RACE	NCAL1	3’ RACE	AATGTCCAAGTGGCTGCAGT
		5’ RACE	CAACACTCTGGTGGTAGTCTGA
NCAL1 localization assay	NCAL1	sense	GCAAGAAACCTCAGGACTTGAG
		antisense	CAACACTCTGGTGGTAGTCTGA
	U6	sense	GTGCTCGCTTCGGCAGCACATATAC
		antisense	ATATGGAACGCTTCACGAATTTGCG
	β-Actin	sense	CAGGTCATCACCATTGGCAATGAGC
		antisense	CGGATGTCCACGTCACACTTCATGA
RNA fluorescence *in situ* hybridization	NCAL1	RNA probe	CAACACTCTGGTGGTAGTCTGAATTTAGTAAATATTCCCAGTCATCATCAGATCCAGTCTCGGAAGGCTTTTTTGGGCGCAATGACAGGGGAAAGTGATCCTTCAAAGTCAAACATGTTGCTGTTACTCAAGTCCTGAGGTTTCTTGC

### Detection of protein-coding ability

Full-length NCAL1 was cloned into the pCDH-CMV-MCS-copGFP vector (CD511B-1) with an N-terminal start codon (ATG) and 3×Flag tag. The plasmid was transfected into HEK293T cells as previously described (20). Flag fusion GFP served as a positive control. After 48 h, immunoblotting was performed to detect the Flag tag (cat: 14793; Cell Signaling Technology, Danvers, MA, USA).

### RNA fluorescence *in situ* hybridization

RNA fluorescence *in situ* hybridization was performed as previously reported (21). Briefly, NK92MI cells were fixed on glass slides by centrifugation. The cells were washed twice with RNase-free phosphate-buffered saline (PBS). Cells on the glass slide were fixed using 4% paraformaldehyde in PBS on ice for 10 min. Freshly prepared 0.5% TritonX-100 was added to the slide, which was incubated on ice for 10 min. After dehydration and drying with ethanol, 10 µL of denatured digoxigenin-labeled RNA probe (50 ng total) was added dropwise onto the slide. The probe contains exon 4 and parts of exons 3 and 5 of NCAL1 (**Supplementary Figure 1C**). Then, the slide was placed in a wet box and hybridized overnight at 42°C. After washing, an anti-digoxin-fluorescein antibody (cat:11207741910; Roche Diagnostics, Mannheim, Germany) was added and incubated for 4 h. The slides were washed three times with PBS for 5 min each. DAPI (20 ng/mL; cat: C1002; Beyotime, Shanghai, China) was added dropwise onto the slide and incubated for 10 min at 24°C. After washing three times with PBS for 5 min each, the slides were imaged using an FV3000 confocal microscope (Olympus, Tokyo, Japan). The probe sequence information is listed in **Table 1**.

### Preparation of cytoplasmic and nuclear fractions

RNA was isolated from the nucleus and cytoplasm of NK92MI cells using a nucleocytoplasmic separation kit (cat: AM1921; Invitrogen, Carlsbad, CA, USA). Polymerase chain reaction (PCR) was performed to detect NCAL1 in the nucleus and cytoplasm. β-Actin and U6 were used as controls. The primer information is listed in **Table 1**.

### Plasmid constructs

NCAL1- and Gab2-overexpressing plasmids were constructed using pCDH-CMV-MCS-EF1-copGFP and were used for lentiviral packaging. HEK293T cells were transfected with these plasmids as previously described (20).

### Lentivirus transfection

Primary NK cells or NK92MI cells were seeded in six-well plates (cat: 703001; NEST Biotechnology Co., Ltd, Wuxi, China) at 500,000 cells/well with 2 mL of medium containing 8 μg/mL polybrene. Cells were infected with different lentiviruses at a multiplicity of infection (MOI) of 10, centrifuged at 2000 rpm for 2 h at 37°C, and cultured at 37°C. The medium was replaced with fresh medium every 2 days, beginning 12–14 h after infection.

### *In vitro* knockdown experiments

A panel of siRNAs for NCAL1 and *Gab2* was designed and synthesized by Viewsolid Biotech (Beijing, China). The primer sequences are listed in **Table 1**. NK92MI cells were transfected with 200 nM siRNAs using Cell Line Nucleofector Kit R (cat: VCA-1003; Lonza, Basel, Switzerland).

### RNA extraction and quantitative PCR

Total RNA was extracted using an RNA extraction kit (cat: K0732; Thermo Fisher Scientific, Waltham, MA, USA). cDNA was obtained using a cDNA synthesis kit (cat: 11123ES10; Yeasen, Shanghai, China). Quantitative PCR (qPCR) was performed using 2×RealStar Green Fast Mixture (cat: A303-05; GenStar, Beijing, China) in a CFX384 Real-Time System C1000 Touch Thermal Cycler (Bio-Rad Laboratories, Hercules, CA, USA). The primers used for qPCR are listed in **Table 1**.

### RNA sequencing

NK92MI cells overexpressing NCAL1 were sequenced at the Novogene Bioinformatics Institute (Beijing, China).

### CD107a degranulation assay

NK92MI and K562 cells were incubated at a ratio of 5:1 at 37 °C in 5% CO_2_. Mouse monoclonal antibody against human CD107a-APC (5 μL/test, cat: 560664, lot: 9183690; BD Biosciences) or isotype control antibody was added to the cells. After incubation for 1 h, 0.1% GolgiStop (cat: 555028; BD Biosciences) was added. After another 3 h of incubation, the cells were collected and stained with mouse monoclonal antibodies against human CD56-FITC (5 μL/test, cat: 562794, lot: 0010250; BD Biosciences). The cells were washed once with PBS and analyzed using a FACSAria II flow cytometer (BD Biosciences). The data were analyzed using FlowJo software version 10 (Tree Star, Inc., Ashland, OR, USA).

### Cytotoxicity assay of NK cells

NK cell-mediated cytotoxicity was analyzed by performing a calcein-release test as previously described (22). Briefly, 100 μL of K562 (target) cells labeled with calcein-AM (cat: C326; Dojindo Laboratories, Kumamoto, Japan) at a concentration of 5 × 10^4^ cells/mL were added to 96-well plates. Then, 100 μL of NK (effector) cells per well were added at effector-to-target cell ratios of 1.25:1, 2.5:1, 5:1, and/or 10:1. Minimum release was achieved by incubating the target cells in medium alone, and maximum release was achieved after treatment with 0.46% Triton X-100. All experiments were performed in triplicate. The plates were incubated at 37°C in 5% CO_2_. After 4 h, 100 μL of supernatant was transferred into black 96-well plates and evaluated using a Synergy HT Microplate Reader (BioTek Instruments, Winooski, VT, USA). Cytotoxicity was calculated using the following formula: specific lysis (%) = [(experimental release – minimum release)/(maximum release – minimum release)] × 100%.

### RNA reverse transcription-associated trap

RNA reverse transcription-associated trap (RAT) was performed as previously reported (23, 24). NK92MI cells were fixed with formaldehyde and were treated with a hypotonic solution (10 mM HEPES pH 7.9, 1.5 mM MgCl_2_, 10 mM KCl, 0.4% NP40) to lyse the cell membrane and isolate the nuclei. Reverse transcription was carried out using biotin-labeled dNTP Mix (cat: R0191; Thermo Fisher Scientific) with specific primers complementary to NCAL1 (**Table 1**). The nucleus was lysed by ultrasonication, and biotinylated lncRNA-cDNA/chromatin DNA complexes were pulled down with streptavidin Dynabeads (cat: 11205D; Invitrogen). Protease K (cat: AM2542; Thermo Fisher Scientific) was added to degrade the protein and genomic DNA interacting with NCAL1.

### Methylation analysis by sodium bisulfite sequencing

Genomic DNA from NCAL-overexpressing NK92MI cells was isolated using a DNeasy Blood and Tissue kit (cat: 69504; Qiagen GmbH, Hilden, Germany). The DNA was modified with bisulfate using an EZ DNA Methylation-Gold kit (cat: D5005Z; ZYMO Research, Los Angeles, CA, USA). DNA was amplified using specific primers for *Gab2* (**Table 1**). The PCR products were cloned using a pJET PCR Cloning kit (cat: K1231, Thermo Fisher Scientific). Ten independent clones from each sample were sequenced to determine the DNA methylation status.

### Chromatin immunoprecipitation and qPCR assay

Chromatin immunoprecipitation (ChIP) assays were performed using a Pierce Agarose ChIP Kit (cat: 26156; Thermo Fisher Scientific). Specific trimethyl-H3K4 antibody (10 μL/10 μg chromatin; cat: 9727s, lot: 10; Cell Signaling Technology), acetyl-H3K27 antibody (20 μL/10 μg chromatin; cat: 4353s, lot: 8; Cell Signaling Technology), and Sp1 antibody (5 μL/10 μg chromatin; cat: PA5-29165, lot: RK2287676, Invitrogen) were used to determine the promoter profile of *Gab2*. Normal rabbit IgG was used as a negative control. DNA was extracted and analyzed using qPCR with specific primers (**Table 1**) targeting the *Gab2* promoter. Enrichment was calculated using the following formula: Input % = 2 [Ct (NCAL1/vector group) – (Ct (input of NCAL1/vector group) – Log2 (input dilution factor of NCAL1/vector group))].

### Western blot assay

Proteins from different groups of NK92MI cells were extracted and quantified using a BCA Protein Assay Kit (cat: PA115-02; Tiangen Biotech Co., Ltd., Beijing, China). Western blotting analysis was performed using mouse monoclonal antibodies against pAKT, AKT, PI3K, and pPI3K (dilution=1:1000; cat: 4060S, lot: 16; cat: 9272S, lot: 28; cat: 4257S, lot: 7; cat: 4228S, lot: 5; respectively, Cell Signaling Technology) and β-Actin (cat: AF5003, lot: 123020210325; Beyotime); appropriate secondary antibodies; and an ECL kit (Beijing Labgic Technology Co., Ltd., Beijing, China).

### Statistical analysis

Data were analyzed by two-tailed paired/unpaired *t*-tests and one/two-way analysis of variance (ANOVA) using GraphPad Prism 8 software (GraphPad, Inc., San Diego, CA, USA).

## Results

### Identification of lncRNA NCAL1 as a potential enhancer of NK cell cytotoxicity

**Figure 1:**
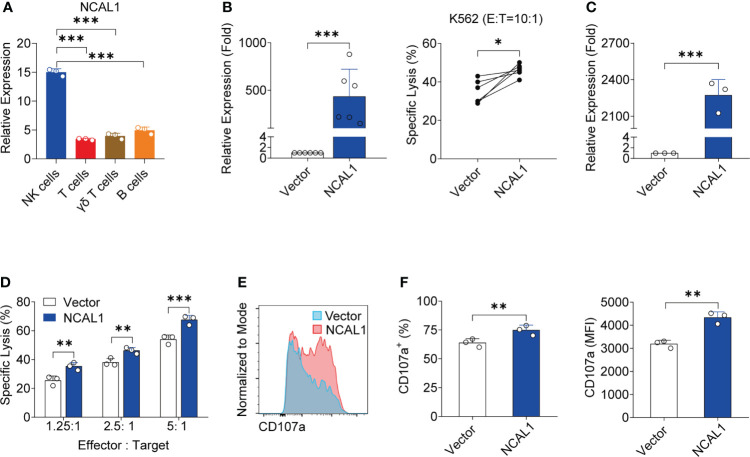
NCAL1 is a novel lncRNA that enhances NK cell cytotoxicity. **(A)** NCAL1 is highly expressed in NK cells. Relative mRNA expression of NCAL1 in human primary NK cells, T cells, γδ T cells, and B cells. Data represent three independent experiments. ***p < 0.001, one-way ANOVA. **(B)** NCAL1 overexpression increases the killing effect of primary NK cells (left panel). Primary NK cells were transfected with NCAL1-overexpressing lentivirus and control vector lentivirus for 48 h. After GFP-positive cell selection, stable cells were collected for qPCR analysis. n = 6, ***p < 0.001, two-tailed paired *t*-test. The right panel shows cytotoxicity following NCAL1 overexpression in primary NK cells, as detected *via* calcein-release assays. n = 6, *p < 0.05, two-tailed paired *t*-test. **(C)** NCAL1 overexpression in NK92MI cells. NK92MI cells were transfected with NCAL1-overexpressing lentivirus and control vector lentivirus. After GFP-positive cell selection, stable cells were collected for qPCR analysis. ***p < 0.001, two-tailed paired *t*-test. **(D)** Cytotoxicity of NK92MI cells overexpressing NCAL1 at various effector cell:target cell ratios, as detected by calcein-release assays. Data represent three independent experiments. **p < 0.01, ***p < 0.001, two-way ANOVA. **(E)** Representative flow cytometry analysis of CD107a expression in NCAL1-overexpressing NK92MI cells. **(F)** CD107a^+^ cell percentages and mean fluorescence intensity (MFI) indicating the CD107a expression in NCAL1-overexpressing NK92MI cells. Data represent three independent experiments. **p < 0.01, two-tailed paired *t*-test.

The molecular factors that control NK cell activity against tumor cells are not completely understood. Using RNA-seq, we identified the lncRNA NCAL1 as a potential factor that correlates with NK cell activity. NCAL1 is located on the short arm of chromosome 2 and has 17 exons (**Supplementary Figure 1A**). Using a cDNA end RACE assay, we characterized the full-length NCAL1 sequence (**Supplementary Figure 1B**). Another lncRNA, CYTOR, is near NCAL1 on chromosome 2 but differs from NCAL1 except for the first exon (**Supplementary Figure 1C**). A lncRNA was considered a novel isoform if it shared some exons with an annotated gene. Therefore, we believe that NCAL1 is a novel lncRNA (GenBank accession number: BankIt2596396 Seq1 ON863928). Since CYTOR is located close to NCAL1, shares a first exon, and has several isoforms, we hypothesized that NCAL1 may also have several isoforms. More importantly, we found that the expression of NCAL1 in primary NK cells was significantly higher than that in the other immune cells (**Figure 1A**).

To explore the functional role of NCAL1, we evaluated the functional relevance of NCAL1 expression in primary NK cells and NK92MI cells. NCAL1 was expressed under the control of the CMV promoter in a lentiviral vector (pCDH). An empty vector was used as the negative control. After lentiviral transfection, primary NK cells were collected for real-time qPCR and cytotoxicity assays. Primary NK cell activity significantly increased when NCAL1 was overexpressed (**Figure 1B**).

Next, we examined whether NCAL1 could activate the killing activity of NK92MI cells. NCAL1 was overexpressed in NK92MI cells using a lentivirus system with GFP. Forty-eight hours post-transfection, most NK92MI cells expressed GFP (**Supplementary Figure 2A**). Then, GFP expression in NK cells was confirmed using flow cytometry, with the proportion of GFP reaching as high as 45.6% (**Supplementary Figure 2B**). When NCAL1 was overexpressed (**Figure 1C**), the killing activity of NK92MI cells was significantly increased at all effector-to-target ratios (1.25:1, 25.49% vs. 35.34%, *p* < 0.01; 2.5:1, 38.13% vs. 46.29%, *p* < 0.01; and 5:1, 53.98% vs. 67.54%, *p* < 0.001) (**Figure 1D**). Moreover, CD107a expression in NCAL1-overexpressing NK92MI cells was significantly higher than that in the control group (74.85% vs. 63.90%, *p* < 0.01; mean fluorescence intensity: 4337 vs. 3188, *p* < 0.01) (**Figures 1E, F**).

### NCAL1 knockdown reduces killing activity of NK cells

**Figure 2:**
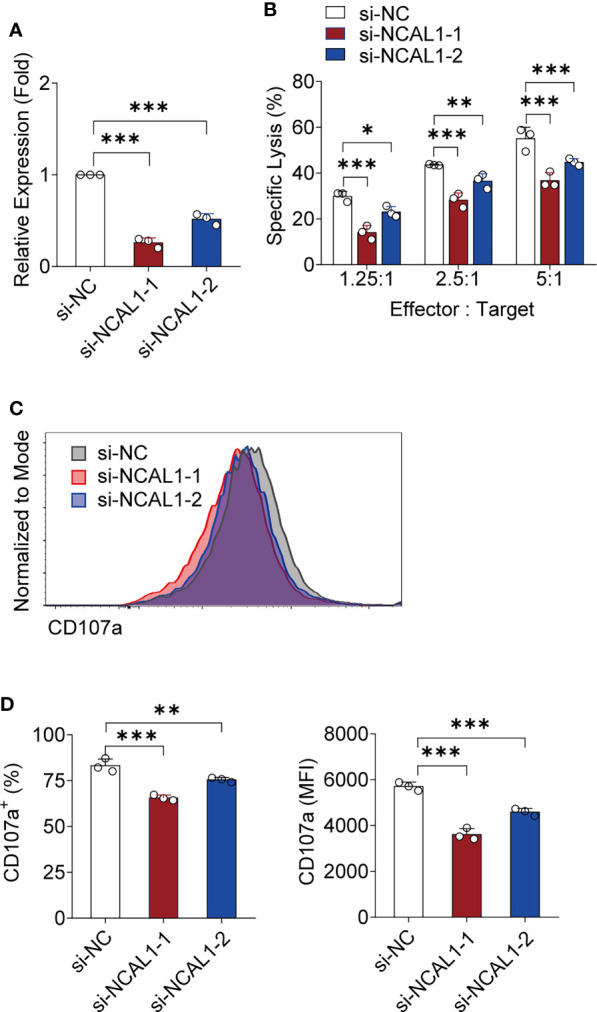
Knocking down NCAL1 reduces NK cell-mediated cytotoxicity. **(A)** siRNA-mediated NCAL1 knockdown in NK92MI cells. si-NCAL1-1 and si-NCAL1-2: siRNAs targeting NCAL1; si-NC: random siRNA control. ***p < 0.001, one-way ANOVA. **(B)** Cytotoxicity mediated by NCAL1-knockdown NK92MI cells at various effector cell:target cell ratios, as detected by calcein-release assays. Data represent three independent experiments. *p < 0.05, **p < 0.01, ***p < 0.001, two-way ANOVA. **(C)** Representative flow cytometry analysis of CD107a expression in NCAL1 knockdown NK92MI cells. **(D)** Statistical analyses of percentages and mean fluorescence intensity (MFI) of CD107a expression in NCAL1 knockdown NK92MI cells. Data represent three independent experiments. **p < 0.01, ***p < 0.001, one-way ANOVA.

To further explore the effect of NCAL1 on NK cell-mediated cytotoxicity, we used small interfering RNA (siRNA) to knock down NCAL1 expression in NK92MI cells. The siRNAs were located in exons 3 and 5 of NCAL1, where NCAL1 can be spliced (**Supplementary Figure 1C**). After knocking down NCAL1 (**Figure 2A**), the ability of NK cells to kill tumor cells decreased at different effector-to-target ratios (1.25:1, 29.91% vs. 14.14%, *p* < 0.001, 29.91% vs. 23.08%, *p* < 0.05; 2.5:1, 43.61% vs. 28.30%, *p* < 0.001, 43.61% vs. 36.60%, *p* < 0.01; 5:1, 55.07% vs. 36.81%, *p* < 0.001, 55.07% vs. 44.80%, *p* < 0.001) (**Figure 2B**). Additionally, CD107a expression in NK92MI cells was decreased after knocking down NCAL1 (**Figures 2C, D**).

### NCAL1 does not code for protein and predominantly resides in the nucleus

**Figure 3:**
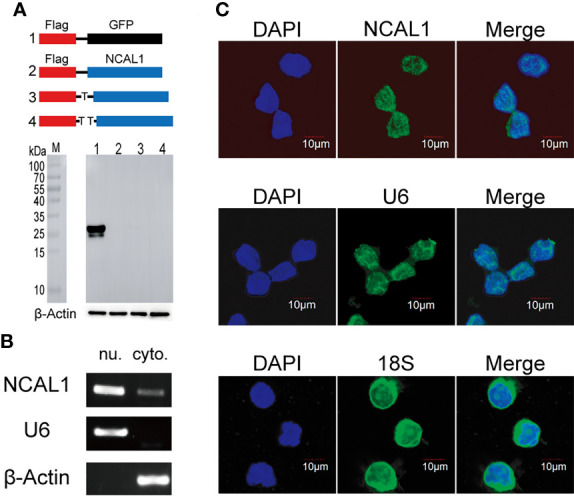
NCAL1 has no coding potential and mainly resides in the nucleus. **(A)** NCAL1 has no coding capability. Full-length NCAL1 was cloned into the pCDH vector with the N-terminal start codon ATG and 3×Flag tag in three coding possibilities. Then, the plasmid was transfected into HEK293T cells. Flag-tagged GFP was used as a positive control. After 48 h, immunoblotting was used to detect the Flag tag. **(B)** NCAL1 localization assays were performed using cytoplasmic and nuclear separation and PCR. Cytoplasmic (cyto.) and nuclear (nu.) RNA was isolated and reverse-transcribed into cDNA. The location of NCAL1 was determined using PCR. U6: nuclear control. β-Actin: cytoplasm control. **(C)** RNA fluorescence *in situ* hybridization to detect NCAL1 in NK92MI cells. U6: nuclear control. 18S: cytoplasm control.

Most lncRNAs do not encode proteins. To examine the protein-coding function of NCAL1, full-length NCAL1 was cloned into the pCDH vector with the N-terminal start codon ATG and Flag tag. The plasmid was then transfected into HEK293T cells. After 48 h, immunoblotting was performed to detect the Flag tag, which confirmed that NCAL1 had no protein-coding function (**Figure 3A**). lncRNA functions are associated with their subcellular localization (25). Cytoplasmic and nuclear RNA isolation assays and RNA fluorescence *in situ* hybridization indicated that NCAL1 was primarily located in the nucleus (**Figures 3B, C**).

### NCAL1 facilitates NK cell cytotoxicity by upregulating Gab2

**Figure 4:**
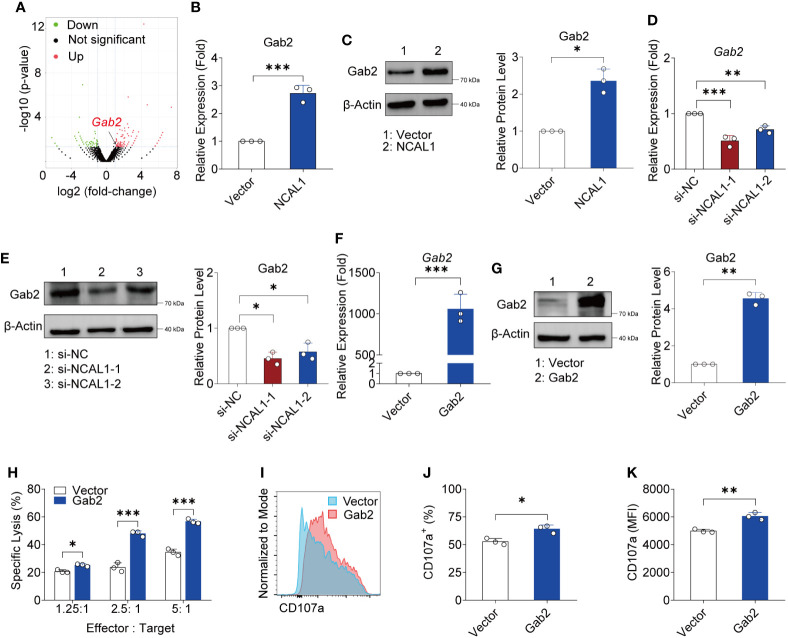
NCAL1 increases NK cell-mediated cytotoxicity by upregulating Gab2. **(A)** Volcano plot of differentially expressed genes between NCAL1-overexpressing NK92MI cells and control NK92MI cells. The red dots on the right indicate upregulated genes, and the green dots on the left indicate downregulated genes. **(B)** NCAL1 overexpression increases Gab2 gene expression. NK92MI cells were transfected with NCAL1-overexpressing lentivirus or control vector lentivirus for 48 h. After GFP-positive cell selection, stable cells were collected for qPCR to quantify Gab2. ***p < 0.001, two-tailed paired *t*-test. **(C)** NCAL1 overexpression enhances Gab2 protein expression. Immunoblotting was used to detect Gab2 protein. A representative western blot is shown on the left side. The right side shows the quantification of the bands from three independent experiments. **(D)** Knockdown of NCAL1 decreases *Gab2* gene expression. NK92MI cells were transfected with NCAL1 siRNA for 48 h. mRNA from transfected NK92MI cells was used to quantify *Gab2*. si-NCAL1-1 and si-NCAL1-2: siRNAs targeting NCAL1; si-NC: random siRNA control. **p < 0.01, ***p < 0.001, one-way ANOVA. **(E)** NCAL1 knockdown decreases Gab2 protein expression. Immunoblotting was used to detect Gab2 protein. A representative western blot is shown in the left panel. The right panel shows the quantification of the bands from three independent experiments. **(F–K)** NK92MI cells were transfected with Gab2 overexpression lentivirus or control vector lentivirus. After GFP-positive cell selection, stable cells were collected. **(F)** *Gab2* gene expression in NK92MI cells. Data represent three independent experiments. ***p < 0.001, one-way ANOVA. **(G)** Immunoblotting was used to detect Gab2 protein. A representative western blot is shown in the left panel. The right panel shows the quantification of the bands from three independent experiments. **p < 0.01, by two-tailed paired t-test. **(H)** Cytotoxicity induced by Gab2-overexpressing NK92MI cells, as detected *via* calcein-release assays. Data represent three independent experiments. *p < 0.05, ***p < 0.001, two-way ANOVA. **(I)** Representative flow cytometry analysis of CD107a expression in Gab2-overexpressing NK92MI cells. **(J, K)** Statistical analyses of percentages and mean fluorescence intensity (MFI) of CD107a expression in Gab2-overexpressing NK92MI cells. Data represent three independent experiments. *p < 0.05, **p < 0.01, two-tailed paired *t*-test.

To explore the mechanism by which NCAL1 regulates NK activity, we collected NCAL1-overexpressing NK92MI cells and used RNA-seq to identify the downstream targets of NCAL1 in NK cells. NCAL1 overexpression in NK92MI cells upregulated 162 genes and downregulated 75 genes. Differentially expressed genes that are possibly related to NK cell function were selected as candidate genes. qPCR was performed to verify the candidate genes. qPCR results showed that NCAL1 overexpression significantly upregulated Gab2 (**Supplementary Figure 3**; **Figure 4A**). Gab2 gene and protein expression were examined using qPCR and western blotting. We found that Gab2 mRNA and protein expression were significantly increased by NCAL1 overexpression (**Figures 4B, C**). Consistently, siRNA-mediated NCAL1 knockdown significantly decreased Gab2 mRNA and protein expression (**Figures 4D, E**). These results indicate that NCAL1 positively regulates Gab2 gene expression and increases its protein expression.

To evaluate the crosstalk between Gab2 and NK cell cytotoxicity, a Gab2-overexpressing plasmid was constructed and transfected into NK92MI cells using a lentivirus transfection system. Gab2 mRNA and protein were successfully expressed in NK92MI cells (**Figures 4F, G**). We observed that NK92MI cells overexpressing Gab2 showed a stronger killing effect on tumor target cells compared to the control group at different effector-to-target ratios (**Figure 4H**). Furthermore, CD107a expression in Gab2-overexpressing NK92MI cells was significantly higher than that in the control group (**Figures 4I–K**). These results indicate that Gab2 expression is positively correlated with NK cell-mediated cytotoxicity.

### NCAL1 binds to the *Gab2* promoter and epigenetically regulates Gab2 expression

**Figure 5:**
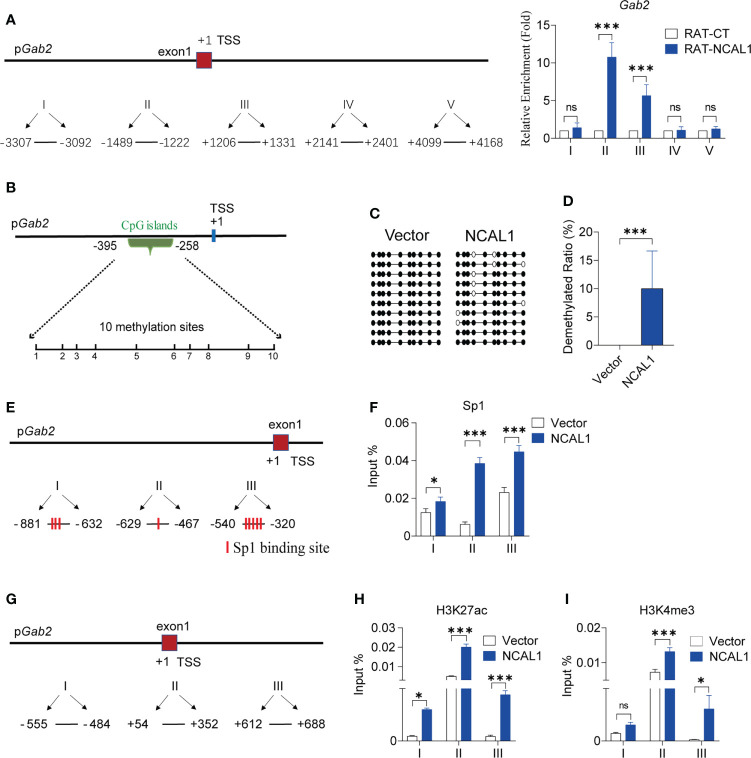
NCAL1 binds to the *Gab2* promoter and epigenetically enhances Gab2 expression. **(A)** Quantitative real-time PCR mapping of NCAL1 binding to the *Gab2* promoter. RAT pulldown assays were used to map NCAL1 binding. Note the enriched NCAL1 binding signals in the *Gab2* promoter region (II, III). Data represent three independent experiments. ns: not significant; ***p < 0.001, two-way ANOVA. RAT-CT: RAT negative control. **(B)** Schematic diagram of CpG islands in *Gab2* promoter. Vertical lines: methylation sites. **(C)** DNA methylation of the *Gab2* promoter in NCAL1-overexpressing NK92MI cells. Open circles: unmethylated CpGs; solid circles: methylated CpGs. **(D)** Statistical analysis of demethylated Gab2 promoters in NCAL1-overexpressing NK92MI cells. ***p < 0.001, two-tailed unpaired *t*-test. **(E)** Quantitative real-time PCR analysis of Sp1 binding sites in the *Gab2* promoter. Three pairs of primers were designed around Sp1 transcription factor binding sites in the *Gab2* promoter. **(F)** Enrichment of *Gab2* DNA sequences for Sp1 occupancy at the *Gab2* promoter of NCAL1-overexpressing NK92MI cells was measured using ChIP-qPCR. Data were obtained from three independent experiments. *p < 0.05, ***p < 0.001, two-way ANOVA. **(G)** Quantitative real-time PCR analysis of the transcription start site near the *Gab2* promoter. Three pairs of primers were designed around the transcription start position of the *Gab2* gene. **(H, I)** Enrichment of *Gab2* DNA sequences for H3K27ac and H3K4me3 occupancy at the *Gab2* promoter of NCAL1-overexpressing NK92MI cells was measured using ChIP-qPCR. Data were obtained from three independent experiments. ns, not significant; *p < 0.05, ***p < 0.001, two-way ANOVA.

To characterize the role of NCAL1 in Gab2 regulation, we mapped NCAL1 targets genome-wide using a RAT approach (**Supplementary Figure 4**). NK92MI cells were collected, and NCAL1 was labeled *in situ* with biotin-dCTP using stringent reverse transcription with three NCAL1-specific complementary primers. Random primers were used as negative controls. Then, biotin–NCAL1 cDNA chromatin complexes were isolated using streptavidin beads. We observed DNA binding to NCAL1 using RAT. We then designed primers targeting the regions upstream and downstream of the *Gab2* transcriptional start site (TSS) and performed qPCR. DNA enrichment was mainly concentrated at the *Gab2* TSS (Sites II and III in **Figure 5A**), suggesting that NCAL1 may epigenetically regulate the *Gab2* promoter.

Using the UCSC database (http://genome.ucsc.edu/), we identified methylation islands in the *Gab2* promoter region (**Figure 5B**). NCAL1 significantly induced *Gab2* promoter demethylation (**Figures 5C, D**). There were several potential Sp1 transcription factor binding sites around the CpG islands in the *Gab2* promoter region, as predicted by the Alibaba 2.1 software (http://gene-regulation.com/pub/programs/alibaba2/index.html) (**Figure 5E**). Therefore, we investigated whether NCAL1 affected the binding of Sp1 to the *Gab2* promoter. ChIP-qPCR indicated that NCAL1 substantially increased Sp1 binding to potential binding sites around the CpG islands of the *Gab2* promoter (**Figure 5F**). These data suggest that NCAL1 promotes Gab2 expression by inducing DNA demethylation and promoting Sp1 binding at the *Gab2* promoter.

Furthermore, we determined that H3K27ac and H3K4me3 were enriched at the TSS of Gab2, based on the data from the UCSC database. To determine the effect of NCAL1 on H3K27ac and H3K4me3 enrichment at the *Gab2* promoter, NCAL1 was overexpressed in NK92MI cells. ChIP was carried out using antibodies against H3K27ac and H3K4me3. Three pairs of primers around the TSS of *Gab2* were selected (**Figure 5G**), and qPCR was performed to detect H3K27ac and H3K4me3 near the *Gab2* TSS. NCAL1 enhanced H3K27ac and H3K4me3 levels at the *Gab2* TSS (**Figures 5H, I**). Collectively, these results suggest that NCAL1 promotes *Gab2* gene expression by modifying the epigenotypes in the *Gab2* promoter.

### NCAL1 and Gab2 cooperatively activate the PI3K-AKT signaling pathway

**Figure 6:**
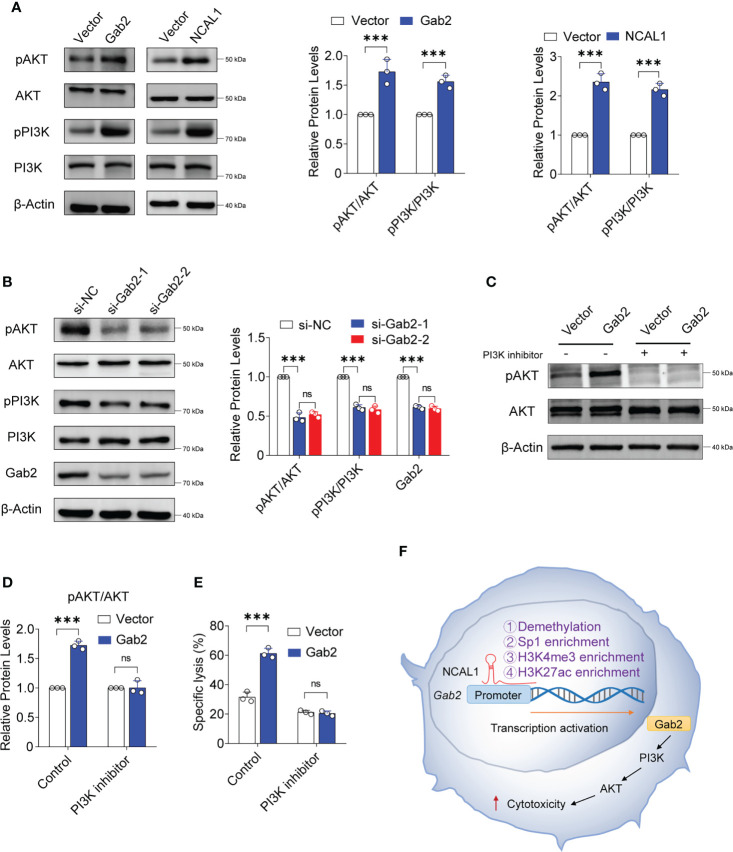
NCAL1 functions through the Gab2-PI3K-AKT axis. **(A)** NK92MI cells were transfected with Gab2 overexpression lentivirus, NCAL1 overexpression lentivirus, or control vector lentivirus. After GFP-positive cell selection, total protein extracts were obtained for immunoblotting. Representative western blots are shown in the left panel. The right panel shows the quantification of the bands from three independent experiments. Gab2: Gab2 overexpression; NCAL1: NCAL1 overexpression. **(B)** siRNA-mediated Gab2 knockdown in NCAL1-overexpressing NK92MI cells. Total protein extracts were obtained for immunoblotting. si-Gab2-1 and si-Gab2-2: siRNAs targeting Gab2; si-NC: random siRNA control. **(C–E)** NK92MI cells were transfected with Gab2 overexpression lentivirus or control vector lentivirus. After GFP-positive cell selection, the cells were treated with a PI3K inhibitor for 24 h. Total protein extracts were obtained for immunoblotting. **(C)** Representative western blots. **(D)** Quantification of the bands from three independent experiments. **(E)** Cytotoxicity was detected *via* calcein-release assays. Data represent three independent experiments. ns: not significant; ***p < 0.001, two-way ANOVA. **(F)** A proposed model describing how NCAL1 enhances the cytotoxicity of NK cells.

To analyze the mechanism by which NCAL1 regulates NK92MI cell-mediated cytotoxicity, we examined the PI3K-AKT signaling pathway, which is closely related to Gab2 and NK cell activation (26–28). We found that pAKT and pPI3K expression significantly increased after overexpressing NCAL1 and Gab2 (**Figure 6A**). PI3K-AKT activation was weakened when Gab2 was knocked down in NCAL1-overexpressing NK92MI cells (**Figure 6B**). Furthermore, inhibition of PI3K with pictilisib suppressed AKT phosphorylation (**Figure 6C, D**) and inhibited the killing activity of Gab2-overexpressing NK cells (**Figure 6E**). These results suggest that NCAL1 improves the NK cell-mediated cytotoxicity through the Gab2-PI3K-AKT axis.

## Discussion

NK cells are important for natural immunity. These cells are involved in immune surveillance and can identify and eliminate mutated cells in the body, thereby preventing cancer onset and progression (29, 30). lncRNAs perform diverse biological functions (31), such as regulating gene expression at the transcript level, thus participating in many biological processes such as chromatin modification, chromosome silencing, genome imprinting, and transcriptional activation (13, 32–34). Although thousands of lncRNAs have been identified in the human transcriptome (35), most of their functions remain uncharacterized (36). Previously, we discovered that valproic acid could inhibit the killing ability of NK cells (19). NGS was performed to identify lncRNAs in NK cells that are altered by treatment with valproic acid (**Supplementary Figure 5**). By analyzing the NGS data, we identified an unknown lncRNA on chromosome 2 called NCAL1. We observed that NCAL1 expression is higher in NK cells than in the other immune cells. Further experiments indicated that NCAL1 is positively correlated with NK cell-mediated cytotoxicity. Interestingly, after primary NK cells were treated with the S protein of SARS-CoV-2, NCAL1 expression decreased, and NK cell activity toward K562 cells decreased (unpublished data). These results indicate that NCAL1 may play an important role in the killing ability of NK cells.

lncRNA localization in cells is closely related to their function (37). NCAL1 is mainly found in the nucleus, indicating that it regulates NK cell function by modifying gene transcription. The overexpression of NCAL1 in NK cells upregulated Gab2 expression. Gab2 is a scaffold protein with multiple functions in immune cell signaling (38). In mast cells, Gab2 is important for degranulation and cytokine release (27). Further, Gab2 can inhibit T cell receptor-mediated signaling events (39). However, the function of Gab2 in human NK cells remains unclear. NCAL1 may partly enhance the cytotoxicity of human NK cells by upregulating Gab2 expression. Indeed, we found that when Gab2 is overexpressed in human NK cells, NK cell activity toward tumor cells significantly increases, indicating that Gab2 positively regulates the killing activity of human NK cells. However, Gab2 does not disrupt NK cell development and function in mice (40). Considering the striking differences in Gab2 functions between human and mouse NK cells, our findings suggest that the role of Gab2 is species-specific.

Gab2 contains multiple tyrosine phosphorylation sites (41). After phosphorylation, these sites can recruit signaling molecules, including molecules that contain SH2 domains, mainly SHP2 and PI3K (28). We found that the PI3K-AKT pathway is activated when either Gab2 or NCAL1 is overexpressed in NK cells. PI3K-AKT axis activation is closely related to the cytotoxicity of NK cells (26, 42). The activation of PI3K-AKT was suppressed when Gab2 was knocked down in NCAL1-overexpressing NK92MI cells. A PI3K inhibitor suppressed AKT phosphorylation and inhibited the cytotoxicity of NK cells overexpressing Gab2. Therefore, NCAL1 may activate the PI3K-AKT axis and increase the cytotoxicity of NK cells by upregulating Gab2 expression. Furthermore, we found that NCAL1 binds to the Gab2 promoter and promotes its demethylation. However, further studies are needed to determine how NCAL1 induces *Gab2* promoter demethylation. Our results predict several potential Sp1 transcription factor-binding sites and enriched H3K27ac and H3K4me3 levels in the *Gab2* promoter region. ChIP experiments showed that NCAL1 enriched Sp1, H3K4me3, and H3K27ac levels at the Gab2 promoter. These results indicate that NCAL1 binds to the Gab2 promoter region and regulates Gab2 gene transcription through an epigenetic mechanism (**Figure 6F**). Therefore, we speculate that NCAL1 might reduce *Gab2* methylation, which loosens the associated chromatin and promotes increased Sp1, H3K27ac, and H3K4me3 levels.

In conclusion, our study identified a new lncRNA NCAL1 that is highly enriched in and is positively related to the cytotoxicity of NK cells. NCAL1 increases the killing activity of NK cells by enhancing Gab2 expression, which subsequently activates the PI3K-AKT pathway. These results indicate that NCAL1 may serve as a potential target to improve the killing ability of NK cells and the efficacy of NK cell therapy.

## Data availability statement

The original contributions presented in the study are included in the article/**Supplementary Material**. The sequencing data presented in the study are deposited in the GEO repository, accession number GSE211801. Further inquiries can be directed to the corresponding authors.

## Ethics statement

The studies involving human participants were reviewed and approved by The Ethics Committee of the First Hospital of Jilin University. The patients/participants provided their written informed consent to participate in this study.

## Author contributions

JC, XinZ, J-FH, and YW conceived and supervised the study. CN and ML designed and carried out part of the experiments and drafted the entire manuscript. XiaZ, YC, SZ, LZ, ZL, and JX carried out part of the experiments. All authors contributed to the article and approved the submitted version.

## Funding

This work was supported by grants from the National Key Research and Development Program of China (grant numbers 2020YFA0707704 and 2018YFA0106902), the Innovative Program of the National Natural Science Foundation of China (82050003), the National Natural Science Foundation of China (grant numbers 31700764, 81972684, 81922055, 31871297, 81874052, and 81702589), a project funded by the China Postdoctoral Science Foundation (grant number 2021M691208), the Jilin Provincial Science and Technology Department (grant numbers 20200201180JC, 20210303002SF, 20200602032ZP, and 20190303146SF), the Jilin Provincial Education Department (grant number JJKH20221060KJ), the Jilin Provincial Development and Reform Commission (grant number 2021C10), the Changchun Science and Technology Bureau (grant number 21ZGY28), and the China Guanghua Foundation & First Hospital of Jilin University (grant numbers JDYYGH2019004 and JDYYGH2019012).

## Conflict of interest

The authors declare that the research was conducted in the absence of any commercial or financial relationships that could be construed as a potential conflict of interest.

## Publisher’s note

All claims expressed in this article are solely those of the authors and do not necessarily represent those of their affiliated organizations, or those of the publisher, the editors and the reviewers. Any product that may be evaluated in this article, or claim that may be made by its manufacturer, is not guaranteed or endorsed by the publisher.

## Supplementary material

Supplementary Figure 1 Genomic location and gene structure of NCAL1. **(A)** Schematic diagram of the chromosome location and structure of NCAL1. **(B)** NCAL1 sequences. **(C)** Comparison between NCAL1 and CYTOR. Also shown is a schematic diagram of the RNA fluorescence probe and si-NCAL1 position.

Supplementary Figure 2 NCAL1 overexpression in NK92MI cells. **(A)** Representative images of NCAL1 overexpression in NK92MI cells. Cells were transfected with lentiviruses and were examined using light and fluorescence microscopy. **(B)** Representative flow cytometry analysis of GFP-positive NK92MI cells. NK92MI cells were transfected with lentiviruses with GFP for 48 h. GFP-positive cells were detected by flow cytometry.

Supplementary Figure 3 Screening of genes regulated by NCAL1. NK92MI cells were transfected with NCAL1-overexpressing lentivirus or control vector lentivirus for 48 h. After GFP-positive cell selection, stable cells were collected for qPCR to quantify candidate genes regulated by NCAL1.

Supplementary Figure 4 Schematic diagram of RNA reverse transcription-associated trap (RAT) qPCR assay. NCAL1 was *in situ* reverse-transcribed using three NCAL1-specific complementary primers containing biotin-dCTP. Random primers were used as negative controls (RAT-CT). After nuclear lysis, the biotin-NCAL1 cDNA chromatin complexes were isolated using streptavidin beads. NCAL1-interacting target DNAs were isolated for quantitative real-time PCR.

Supplementary Figure 5 Schematic diagram of NCAL1 discovery. NK cells were separated from total human PBMCs using an NK cell isolation kit, activated by IL-2 and IL-15 for 24 h, and treated with valproic acid for 24 h. NK cells were collected and the RNA was extracted. Next-generation sequencing (NGS) and qPCR were performed to obtain NCAL1.

## Abbreviations

NK, natural killer; lncRNA, long noncoding RNA; NCAL1, NK cell activity-associated lncRNA 1; PBS, phosphate-buffered saline; RAT, RNA reverse transcription-associated trap; ChIP, chromatin immunoprecipitation; qPCR, quantitative real-time PCR; siRNA, small interfering RNA.
