# Supplementary figures and images for: LncRNA NCAL1 potentiates natural killer cell cytotoxicity through the Gab2-PI3K-AKT pathway
Source: Front Immunol. 2022 Sep 28;13:970195. doi: 10.3389/fimmu.2022.970195 (PMC9554105; doi:10.3389/fimmu.2022.970195)

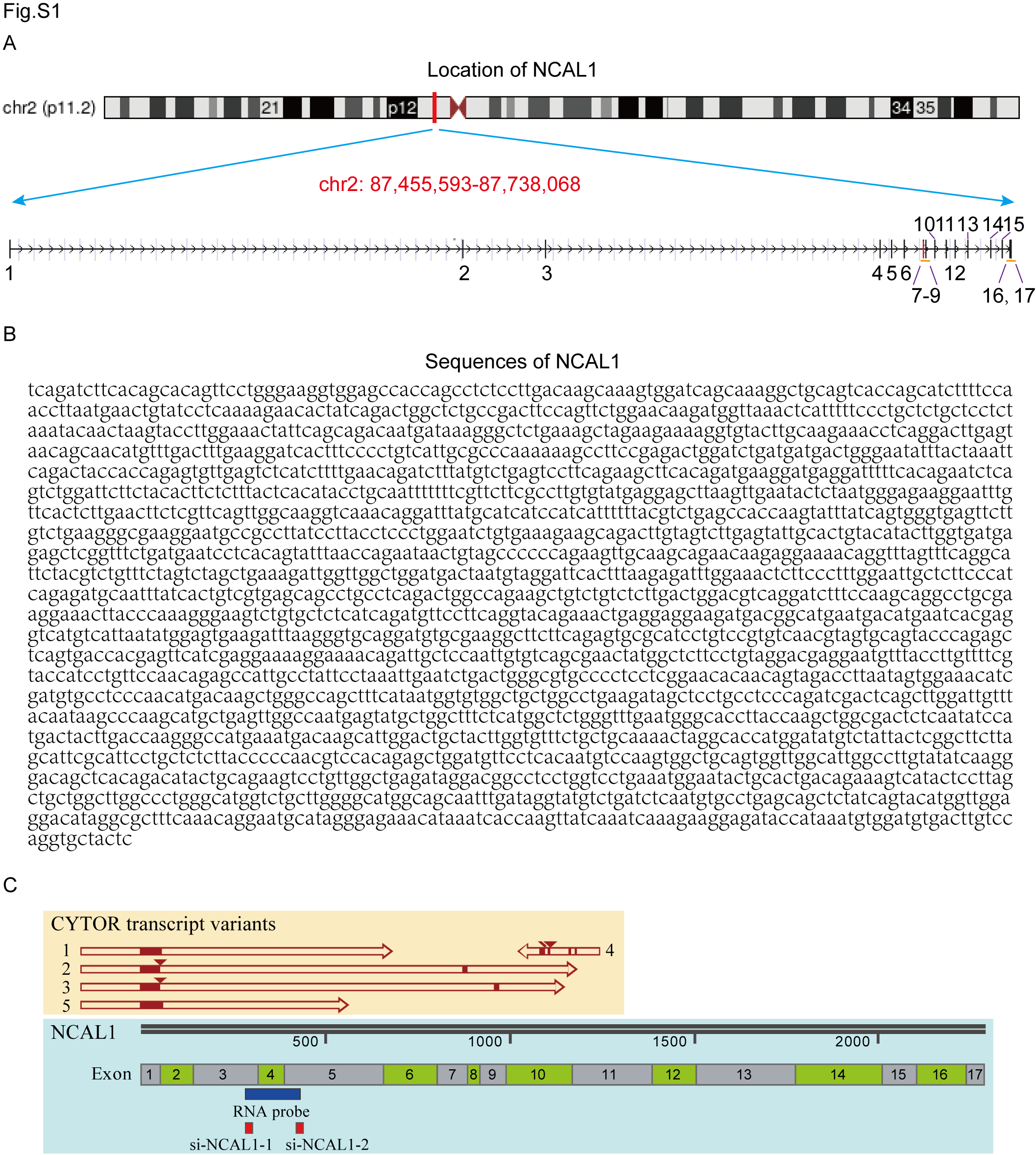

Supplement: Supplementary Figure 1 — Genomic location and gene structure of NCAL1. (A) Schematic diagram of the chromosome location and structure of NCAL1. (B) NCAL1 sequences. (C) Comparison between NCAL1 and CYTOR. Also shown is a schematic diagram of the RNA fluorescence probe and si-NCAL1 position. [file Image_1.tif]

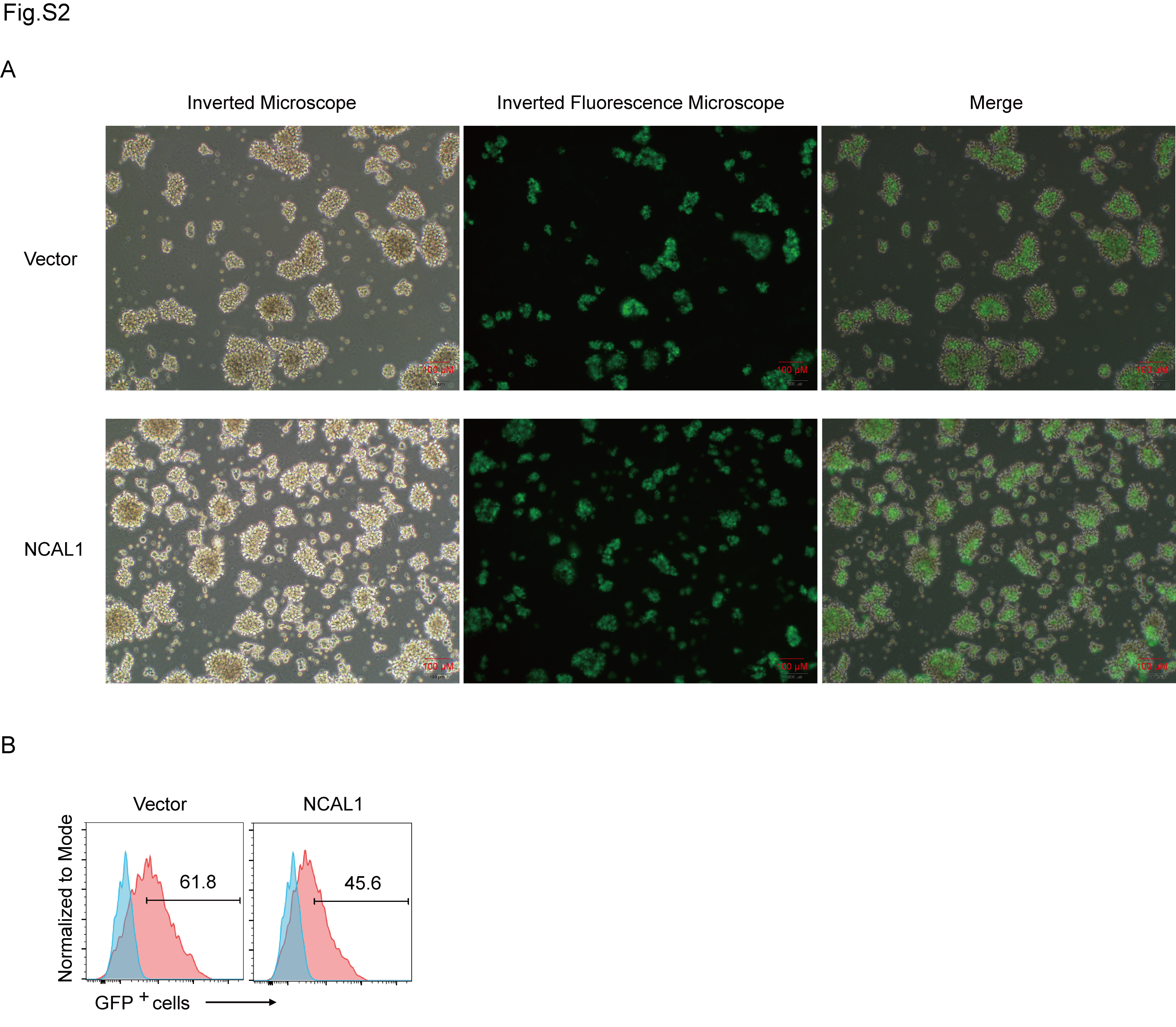

Supplement: Supplementary Figure 2 — NCAL1 overexpression in NK92MI cells. (A) Representative images of NCAL1 overexpression in NK92MI cells. Cells were transfected with lentiviruses and were examined using light and fluorescence microscopy. (B) Representative flow cytometry analysis of GFP-positive NK92MI cells. NK92MI cells were transfected with lentiviruses with GFP for 48 h. GFP-positive cells were detected by flow cytometry. [file Image_2.tif]

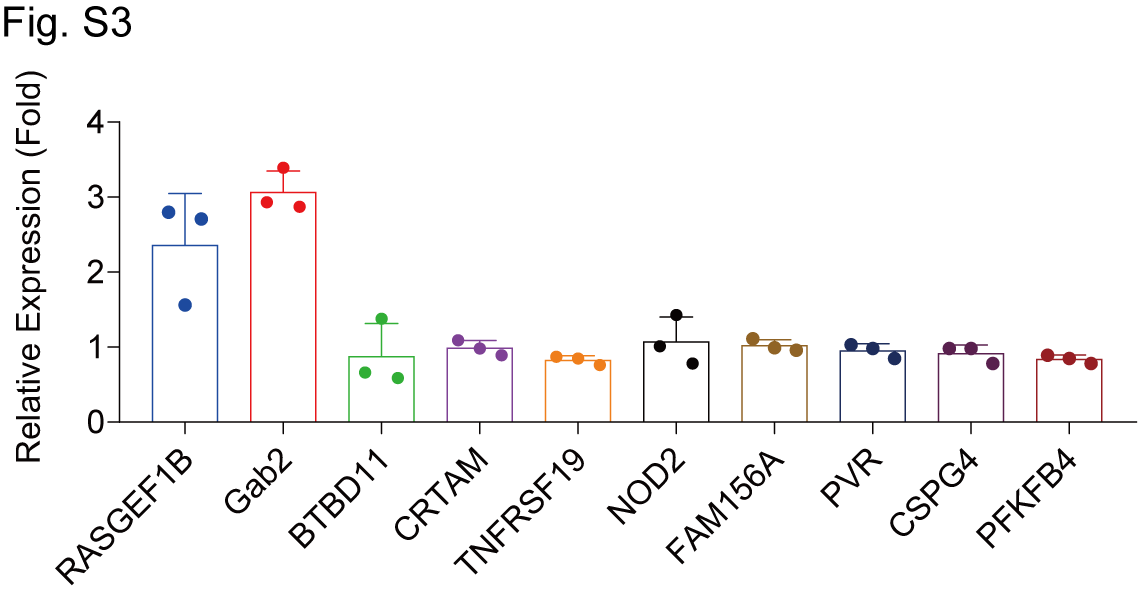

Supplement: Supplementary Figure 3 — Screening of genes regulated by NCAL1. NK92MI cells were transfected with NCAL1-overexpressing lentivirus or control vector lentivirus for 48 h. After GFP-positive cell selection, stable cells were collected for qPCR to quantify candidate genes regulated by NCAL1. [file Image_3.tif]

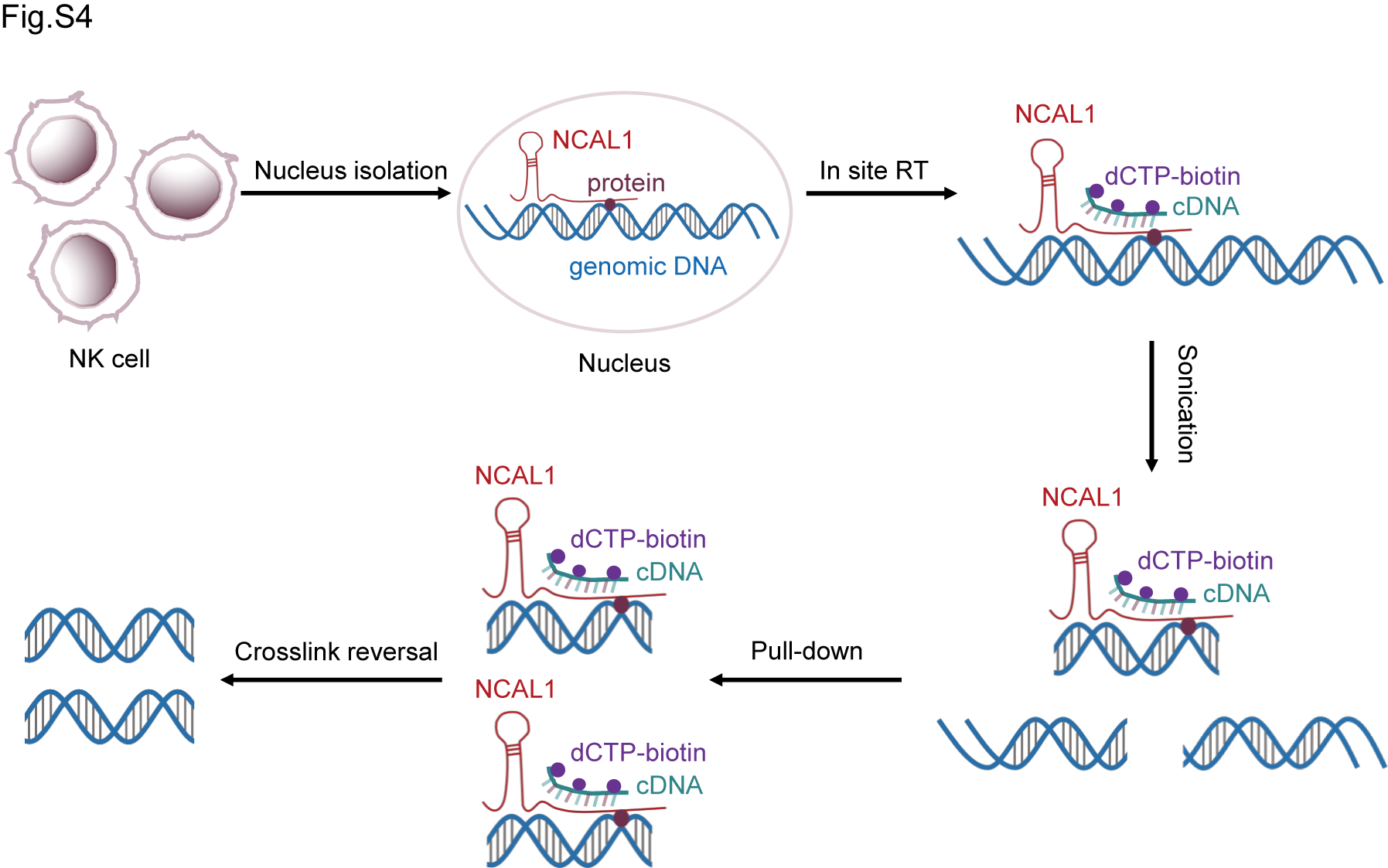

Supplement: Supplementary Figure 4 — Schematic diagram of RNA reverse transcription-associated trap (RAT) qPCR assay. NCAL1 was in situ reverse-transcribed using three NCAL1-specific complementary primers containing biotin-dCTP. Random primers were used as negative controls (RAT-CT). After nuclear lysis, the biotin-NCAL1 cDNA chromatin complexes were isolated using streptavidin beads. NCAL1-interacting target DNAs were isolated for quantitative real-time PCR. [file Image_4.tif]

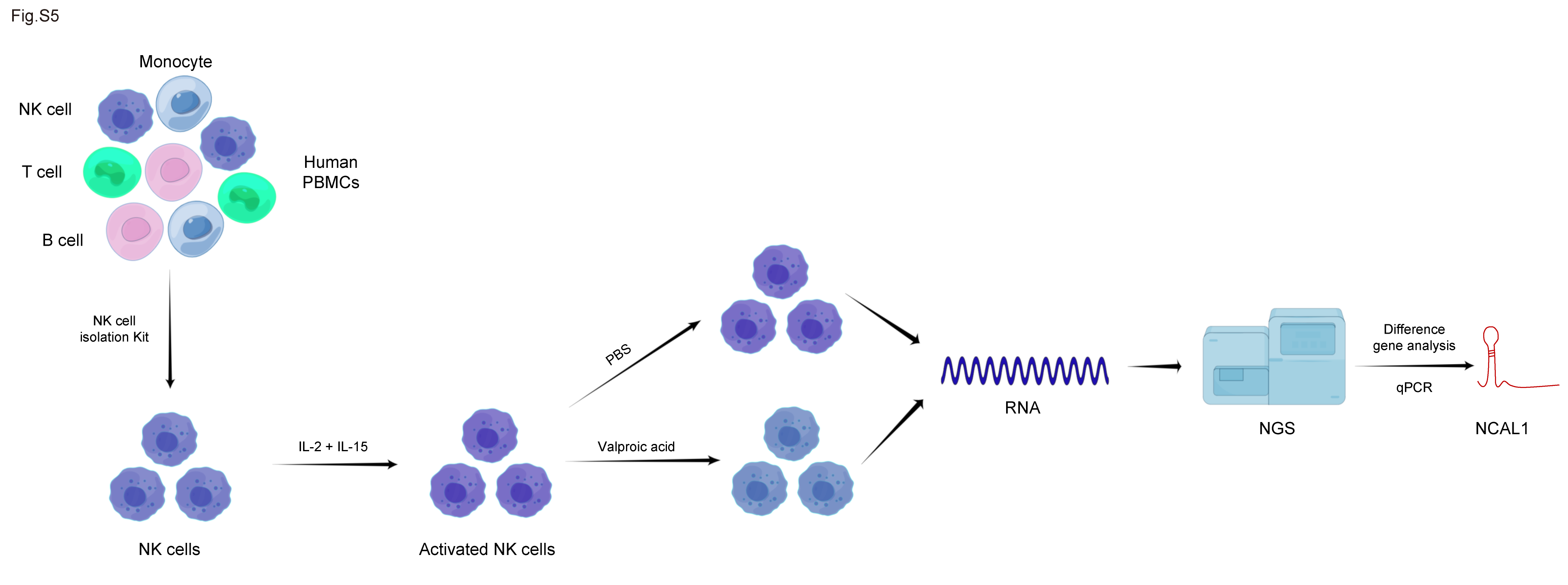

Supplement: Supplementary Figure 5 — Schematic diagram of NCAL1 discovery. NK cells were separated from total human PBMCs using an NK cell isolation kit, activated by IL-2 and IL-15 for 24 h, and treated with valproic acid for 24 h. NK cells were collected and the RNA was extracted. Next-generation sequencing (NGS) and qPCR were performed to obtain NCAL1. [file Image_5.tif]
